# Responses to the correspondence from McDowell *et al*.’s on CAM integrative review of health care professionals in New Zealand

**DOI:** 10.1186/s12906-024-04488-0

**Published:** 2024-05-13

**Authors:** Lizhou Liu, Yong Tang, G. David Baxter, Haiyan Yin, Steve Tumilty

**Affiliations:** 1 Ageing Well National Science Challenge, University of Otago, Dunedin, New Zealand; 2 China-New Zealand Collaboration Centre for Integrative Medicine (CHINZIM), University of Otago, Dunedin, New Zealand; 3 Acupuncture & Tuina School, Chengdu University of Traditional Chinese Medicine, Chengdu, China; 4 China-New Zealand Collaboration Centre for Integrative Medicine (CHINZIM), Chengdu University of Traditional Chinese Medicine, Chengdu, China; 5 Acupuncture & Chronobiology Key Laboratory of Sichuan Province, Chengdu, China; 6 Graduate Research School, University of Otago, Dunedin, New Zealand; 7 Centre for Health, Activity, and Rehabilitation Research, School of Physiotherapy, University of Otago, Dunedin, New Zealand

**Keywords:** Extrapolation, Errors, Survey, Response rate, New Zealand, Complementary and alternative medicine, Physiotherapy, Acupuncture

## Abstract

The authors of the manuscript ‘*Complementary and alternative medicine - practice, attitudes, and knowledge among healthcare professionals in New Zealand: an integrative review’* [1] **disagree with** the assertion by McDowell et al. that our manuscript has extrapolation errors.

First, we disagree with McDowell *et al’*s statement that we intended to extrapolate a very small survey finding to the entire New Zealand physiotherapy population.

The aim of our review [1] was to investigate New Zealand healthcare professionals’ (including physiotherapists as a part of this population) practice of, attitudes toward, and knowledge about complementary and alternative medicine (CAM). Of the 11 included studies in this review, there was only one study (i.e. McDowell et al.) [2] evaluating New Zealand physiotherapists’ practice, attitudes, and knowledge of CAM. This single study explored New Zealand physiotherapists’ opinions and practice *exclusively on* acupuncture/dry needling, and *exclusively for* its use during pregnancy. The background of this study has been well described throughout the manuscript. Taking our manuscript in context, the results of our review should be interpreted in the way that reflects the perspectives of the **study respondents** (rather than the whole professional population). The overall conclusions of our review are robust in that these are based on 11 studies of moderate to high quality, and included a total of 2,060 New Zealand healthcare professionals, including general practitioners (GPs), Plunket nurses, midwifes, pharmacists, specialists, and physiotherapists.

Secondly, as acknowledged by McDowell et al., theirs is a small survey study with a very low response rate; consequently, we have addressed the potential limitations of our paper in the last paragraph of the Discussion [1].

It is important to note that the survey response rate was *not reported* in their original paper, neither was the specific population size of their targeted survey participants. This is important insofar as the current assertions by McDowell et al., concerning statistical extrapolation error does not reflect any supporting references in their original paper.

Thirdly, we acknowledge the potential interests of Jillian McDowell and Susan Heather Kohut being executive members and tutors for the Physiotherapy Acupuncture Association of New Zealand. But the current state of acupuncture practice in New Zealand by physiotherapists (as detailed in their correspondence) is not the main focus of our review, which aimed to provide an overview of New Zealand healthcare professionals’ practice of, attitudes toward, and knowledge about CAM. We would also suggest that the authors’ references to regulation of physiotherapy acupuncture in New Zealand are potentially misleading: firstly acupuncture is regulated for physiotherapy practitioners in New Zealand (it is considered to be part of ‘general scope’); secondly practising within a defined field does not represent some form of advanced or specialist practice as seems to be suggested (these are specific scopes of practice); finally the term ‘physiotherapy acupuncturists’ is not recognised as a protected title or designation by the Physiotherapy Board of New Zealand as the regulator.

## Data Availability

All data generated or analysed during this study are included in the published original article.

## Abbreviations

CAM: complementary and alternative medicine
GPs: general practitioners
